# Gastric Damage and Cancer-Associated Biomarkers in *Helicobacter pylori*-Infected Children

**DOI:** 10.3389/fmicb.2020.00090

**Published:** 2020-02-12

**Authors:** Sergio George, Yalda Lucero, Juan Pablo Torres, Anne J. Lagomarcino, Miguel O’Ryan

**Affiliations:** ^1^Host-Pathogen Interaction Laboratory, Microbiology and Mycology Program, ICBM, Faculty of Medicine, University of Chile, Santiago, Chile; ^2^Department of Pediatrics and Pediatric Surgery, Dr. Roberto del Río Hospital, Faculty of Medicine, Universidad de Chile, Santiago, Chile; ^3^Department of Pediatrics and Pediatric Surgery, Dr. Luis Calvo Mackenna Hospital, Faculty of Medicine, Universidad de Chile, Santiago, Chile; ^4^Millennium Institute on Immunology and Immunotherapy (IMII), Faculty of Medicine, Universidad de Chile, Santiago, Chile

**Keywords:** *Helicobacter pylori*, children, gastric damage, cancer biomarker, oncogene, gastric cancer biomarker

## Abstract

*Helicobacter pylori* (*H. pylori*) is well-known to be involved in gastric carcinogenesis, associated with deregulation of cell proliferation and epigenetic changes in cancer-related genes. *H. pylori* infection is largely acquired during childhood, persisting long-term in about half of infected individuals, a subset of whom will go on to develop peptic ulcer disease and eventually gastric cancer, however, the sequence of events leading to disease is not completely understood. Knowledge on carcinogenesis and gastric damage-related biomarkers is abundant in adult populations, but scarce in children. We performed an extensive literature review focusing on gastric cancer related biomarkers identified in adult populations, which have been detected in children infected with *H. pylori*. Biomarkers were related to expression levels (RNA or protein) and/or methylation levels (DNA) in gastric tissue or blood of infected children as compared to non-infected controls. In this review, we identified 37 biomarkers of which 24 are over expressed, three are under expressed, and ten genes are significantly hypermethylated in *H. pylori*-infected children compared to healthy controls in at least 1 study. Only four of these biomarkers (pepsinogen I, pepsinogen II, gastrin, and SLC5A8) have been studied in asymptomatically infected children. Importantly, 13 of these biomarkers (β-catenin, C-MYC, GATA-4, DAPK1, CXCL13, DC-SIGN, TIMP3, EGFR, GRIN2B, PIM2, SLC5A8, CDH1, and VCAM-1.) are consistently deregulated in infected children and in adults with gastric cancer. Future studies should be designed to determine the clinical significance of these changes in infection-associated biomarkers in children and their persistence over time. The effect of eradication therapy over these biomarkers in children if proven significant, could lead to modifications in treatment guidelines for younger populations, and eventually promote the development of preventive strategies, such as vaccination, in the near future.

## Introduction

*Helicobacter pylori* (*H. pylori*) prevalence rates in adult populations vary from 35 to 90%, depending on the detection method used (serology, urea breath test, stool antigen detection test or invasive methods) and socioeconomic development status of the evaluated population (Hooi et al., 2017). *H. pylori* is epidemiologically relevant due to its causative role in peptic ulcer disease, gastric lymphoma and adenocarcinoma in adults. Annually, nearly one million new cases of gastric cancer (GC) and 738,000 cancer-associated deaths occur worldwide (Karimi et al., 2014) and *H. pylori* is estimated to cause 89% of all GC cases (Plummer et al., 2016). *H. pylori* associated carcinogenesis occurs by both indirect and direct mechanisms. The former are mediated by chronic inflammation, leading to increased cell turnover resulting in an accumulation of mitotic errors and by regulatory T cell induction leading to immune response evasion (Bagheri et al., 2016; Kalisperati et al., 2017). Direct mechanisms are mediated by bacterial factors which affect specific molecular targets on the gastric epithelial cells, resulting in DNA damage (Bagheri et al., 2016). Virulence factors, such as CagA (cytotoxin-associated gene A) and VacA (vacuolating cytotoxin A), can induce mutations in genes that regulate the cell-cycle, deficiencies in DNA repair mechanisms, loss of cell adhesion and epigenetic changes, resulting in deregulation of the cell cycle, proliferation, and malignant transformation (Ahn and Lee, 2015). Epigenetic modifications associated with *H. pylori* include DNA methylation and histone modifications, which may be associated with gastric carcinogenesis (Hanada and Graham, 2014; Shimazu et al., 2015; Valenzuela et al., 2015). Comprehensive reviews on *H. pylori* associated carcinogenesis or gastric damage-related biomarkers in adults have been widely published (Skierucha et al., 2016; Chmiela et al., 2017).

*Helicobacter pylori* infection is mostly acquired during childhood, and prevalence estimates range from 20% to 50% in asymptomatic children ≤5 years of age and 38 to 79% in children >5 years of age, with the probability of persistence varying widely from 49 to 95% (Zabala Torrres et al., 2017). The sequence of events that leads from childhood persistent infection to the development of malignant disease in adulthood is partially understood. A long term process occurs in gastric tissue from premalignant lesions (gastritis, atrophy, intestinal metaplasia and dysplasia) to malignant carcinoma, described as the “Correa cascade,” where atrophic gastritis is the first recognizable step in the development of future cancer; non-atrophic gastritis in contrast may lead to benign lesions (Correa and Piazuelo, 2012). Thus, it is important to elucidate molecular mechanisms by which persistent infection in a subgroup of *H. pylori* infected children may lead to precancerous and cancer lesions in future adulthood.

As this review will synthesize, most studies on clinical outcomes of *H. pylori* infection or the characterization of genes and molecules in gastric tissue related to infection have been conducted in symptomatic children undergoing endoscopy. Symptoms leading to endoscopic examination were not specified in all the studies reviewed. Clinical guidelines such as the Rome criteria are routinely used worldwide for endoscopy indication. Alarm criteria include a family history of peptic ulcer disease, persistent right upper or right lower quadrant pain, dysphagia, odynophagia, persistent vomiting, gastrointestinal blood loss, nocturnal diarrhea, involuntary weight loss, and deceleration of linear growth, all of which may be manifestations of mucosal inflammation (Hyams et al., 2016). Meanwhile, few studies have evaluated clinical outcomes and molecular markers of gastric damage in asymptomatically infected individuals. Studies in both symptomatic and asymptomatic children are required to better understand the pathogen-host interaction, which may or may not be relevant for disease development. Importantly, biomolecular and gene expression dysregulation found in persistently infected children might be playing a role in disease progression and, in some cases, determining an oncogenic process. To our knowledge, comprehensive reviews on *H. pylori* associated gastric damage- or oncogenic-related biomarkers in children are scarce, and only a few have focused on biomarkers and genes dysregulation in infected children. These reviews have focused on inflammatory responses (Ernst and Gold, 1999; Razavi et al., 2015) describing both proinflammatory (Th1, Th17) and regulatory responses (Treg), however other molecules related to tissue damage and/or cancer have not been summarized. Here we discuss the biological function and potential role in carcinogenesis/gastric damage of genes and/or proteins, previously identified in adult carcinogenesis, that are differentially expressed and/or methylated among infected versus non-infected children, according to the published literature. For the purpose of this review, we are using the National Institutes of Health Biomarkers Definitions Working Group’s definition of “biomarker”: “a defined characteristic that is measured as an indicator of normal biological processes, pathogenic processes or responses to an exposure or intervention” (FDA-NIH Biomarker Working Group, 2019). Thus, we considered biomarkers to be methylated genes and expression products (proteins, mRNA or miRNA) from coding genes, where expression level or methylation status was associated with *H. pylori* infection in children and with gastric cancer or gastric damage in adults. Our aim is to determine if there is sufficient evidence associating *H. pylori* infection in symptomatic and asymptomatic children with biomarkers of gastric damage and oncogenesis described in adults.

### Definition of Terms

Population of interest was defined as children (0–18 years old), symptomatic or asymptomatic with *H. pylori* infection determined by any of the following methods: Urea breath test (UBT), stool antigen test, serology, histopathology, culture from gastric biopsy and urease test. Outcome was the evaluation of expression and/or methylation level of a biomarker (proteins or RNA in case of expression level, and gene in case of methylation level assessment) related to gastric cancer in adults. Comparison group were children with no *H. pylori* infection according to the already named diagnostic methods.

### Search Strategy and Study Selection

We conducted a literature search of PubMed^®^ on December 10, 2019 using the following search terms [pylori AND children AND (cancer OR oncogene OR gastric damage)] with filters humans and English language. Titles and abstracts were screened for relevance. Studies published previous to 1999 were excluded. We first excluded manuscripts with the following designs: non-systematic reviews, systematic reviews/meta-analyses, epidemiologic studies (those including information only on prevalence and/or incidence of *H. pylori* infection in different contexts and/or on gastric cancer), bacterial-factor related studies, case reports, clinical guidelines, therapy or vaccine studies, diagnostic method studies, histopathology studies, animal model studies, *in vitro* studies, studies about microbiome, antibiotic resistance, opinion letters, replies and editorials. Articles that did not include an abstract or have full-text availability in English were also excluded. The remaining publications that included information on potential biomarkers were analyzed in-depth. Eligibility criteria were defined as: (1) studies including both *H. pylori* infected and non-infected children, (2) pediatric data clearly differentiated from adult data, (3) studies on biomarkers (genes and/or proteins) related to cell proliferation, gastric damage and/or cancer, and (4) studies that reported biomarker expression levels (as RNA or protein) and/or methylation level. As our focus is on carcinogenesis, articles dedicated exclusively to immune response were excluded (including articles related to polymorphisms in interleukins and innate immune genes, expression levels of proinflammatory and anti-inflammatory cytokines, and gastric infiltration or systemic levels of different immune cells). We added a direct search for the following biomarkers which were not identified in our initial literature search but known to play a role in adult carcinogenesis: pepsinogen, adhesion molecules, chemokines and gastrin. Finally, when a biomarker was selected, an additional search was performed in PubMed^®^ (using terms pylori AND children AND “biomarker name”), applying the same exclusion criteria. Details on the search process ere depicted in Supplementary Figure S1.

### Oncogenic and Gastric Damage-Related Biomarkers Identified in Children

**FIGURE 1:**
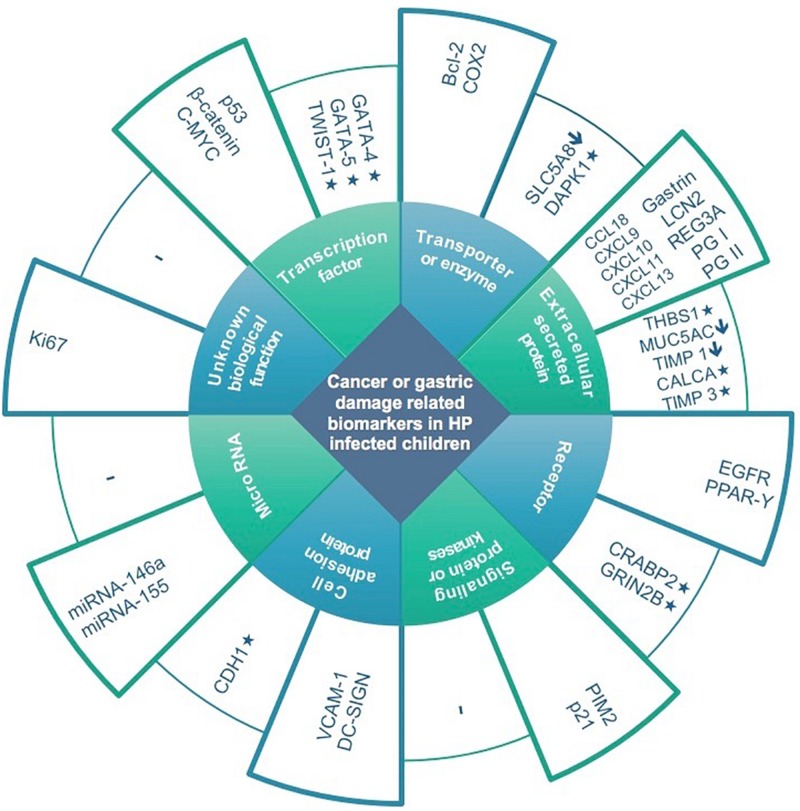
Expression and methylation levels of biomarkers potentially related to gastric damage or oncogenesis in *H. pylori* infected children, classified according to biological function. Higher columns represent overexpression in infected children compared to non-infected controls. Lower columns represent underexpression (↓) and/or methylation (*).

**FIGURE 2:**
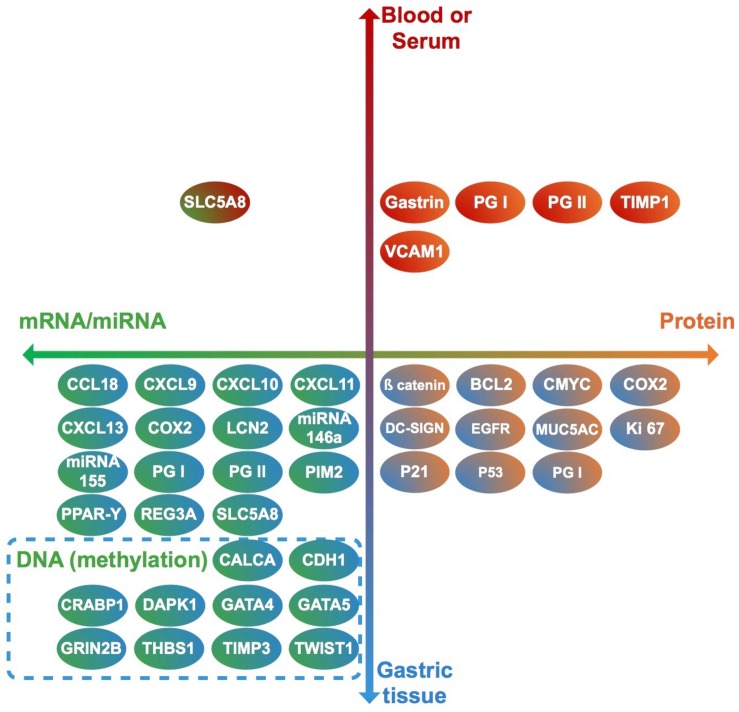
Clinical samples used to analyze each biomarker (vertical axis), and molecules identified in each case (horizontal axis). Each biomarker identified in this review is represented by a circle. Upper half: biomarkers analyzed in blood or serum. Lower half: biomarkers analyzed in gastric tissue. Right half: Biomarkers identified as proteins. Left half: biomarkers identified as RNA or DNA. Genes (DNA) in which methylation levels were analyzed are surrounded by a dashed rectangle.

Thirty-seven studies from 14 different countries were finally selected, involving children and adolescents from 3 months to 19 years of age. Only 1 study included patients older than 18 years (Maciorkowska et al., 2005), but median age were 10–13 years in different groups, so it was no excluded. Most studied children were symptomatic, with only 3/37 studies including asymptomatic children (Supplementary Tables S1, S2). A total of 37 molecules cataloged as biomarkers of carcinogenesis were found to be overexpressed, underexpressed and/or hypermethylated among infected children compared to non-infected controls, as detailed in Tables 1–3 respectively, and summarized in Figure 1. Biomarkers are classified by function (transcription factors, transporters and enzymes, extracellular secreted proteins, signaling protein or kinases, cell adhesion proteins, cell proliferation markers and microRNAs), as shown in Tables 1–3; details on the molecules (proteins, RNA or DNA) analyzed for each biomarker, and sample type (gastric or blood/serum) are depicted in Figure 2; details on the biological function and role in carcinogenesis can be found in Supplementary Table S3. These biomarkers are briefly described below, focusing on the information available in children.

**TABLE 1:** Biomarkers overexpressed in *H. pylori* infected children in at least one study included in this review.

**Biomarker**	**No. of studies with significant upregulation (references)**	**Biological function**	**Potential role in carcinogenesis**
β-catenin	1 (Villarreal-Calderon et al., 2014)	Transcription factor	Oncogenic
BCL-2	1 (Ryszczuk et al., 2016)	Transporter or enzyme	Controversial
C-MYC	1 (Nardone et al., 2001)	Transcription factor	Oncogenic
CCL18	1 (Ikuse et al., 2012)	Extracellular secreted protein	Controversial
CXCL9, 10, 11	1 (Ikuse et al., 2012)	Extracellular secreted protein	Controversial
CXCL13	2 (Ikuse et al., 2012; Obayashi et al., 2016)	Extracellular secreted protein	Oncogenic
COX2*	1 (Kim et al., 2004)	Transporter or enzyme	Controversial
DC-SIGN	1 (Wu et al., 2014)	Cell adhesion protein	Oncogenic
Gastrin**	5 (Kato et al., 2004; Maciorkowska et al., 2006; Plonka et al., 2006; Czaja et al., 2008; Guariso et al., 2009)	Extracellular secreted protein	Oncogenic
EGFR	2 (Maciorkowska et al., 2009; Ryszczuk et al., 2016)	Receptor	Oncogenic
Ki67	4 (Kim et al., 2004; Ozturk et al., 2005; Muñoz et al., 2007; Saf et al., 2015)	Not known	Not known
LCN2	2 (Ikuse et al., 2012; Obayashi et al., 2016)	Extracellular secreted protein	Controversial
p53***	2 (Ozturk et al., 2005; Saf et al., 2015)	Transcription factor	Anti-oncogenic
p21****	1 (Saf et al., 2015)	Signaling protein or kinases	Controversial
Pepsinogen I^$^ *****	8 (Fukuda et al., 2003; Rogers et al., 2003; Lopes et al., 2006; Roma et al., 2006; Guariso et al., 2009; Kassem et al., 2017; Nakayama et al., 2017; Kienesberger et al., 2018)	Extracellular secreted protein	GC biomarker
Pepsinogen II^$^	8 (Fukuda et al., 2003; Rogers et al., 2003; Lopes et al., 2006; De Angelis et al., 2007; Koivusalo et al., 2007; Guariso et al., 2009; Kassem et al., 2017; Nakayama et al., 2017)	Extracellular secreted protein	GC biomarker
PIM2	1 (Obayashi et al., 2016)	Signaling protein or kinases	Oncogenic
PPAR- Υ	1 (Haruna et al., 2008)	Receptor	Controversial
REG3A	1 (Obayashi et al., 2016)	Extracellular secreted protein	Controversial
miRNA-146a	1 (Cortés-Márquez et al., 2018)	Post-transcriptional gene expression regulator	Controversial
miRNA-155	1 (Cortés-Márquez et al., 2018)	Post-transcriptional gene expression regulator	Controversial
VCAM-1	1 (Maciorkowska et al., 2005)	Cell adhesion protein	Oncogenic

*1 study shows no significant mRNA upregulation in infected children. (Haruna et al., 2008). **2 studies show no significant change in expression (Nardone et al., 2001; Villarreal-Calderon et al., 2014). ***5 studies show no significant change in expression (Fukuda et al., 2003; Roma et al., 2006; Xie et al., 2006; De Angelis et al., 2007; Koivusalo et al., 2007), and 1 study show significant downregulation (Lopes et al., 2006); as most studies agree that gastrin is upregulated, it was not included in Table 2. ****1 study published before 1999 shows no significant change in expression (Jones et al., 1997). *****2 studies show no differences in serum levels compared to non-infected controls (De Angelis et al., 2007; Koivusalo et al., 2007). ^ $^1 study shows significant downregulation in gastric tissue (Ikuse et al., 2012). As most studies agree that pepsinogen is upregulated, it was not included in Table 2. No.: Number.

**TABLE 2:** Biomarkers underexpressed in *H. pylori* infected children in at least one study included in this review.

**Biomarker**	**No. of studies with significant downregulation (references)**	**Biological function**	**Potential role in carcinogenesis**
MUC5AC	1 (Park et al., 2015)	Extracellular secreted protein	Controversial
SLC5A8	2 (O’Ryan et al., 2015*, Orellana et al., 2016)	Transporter or enzyme	Anti-oncogenic
TIMP1	1 (Rautelin et al., 2010)	Extracellular secreted protein	Controversial

No.: Number. *Microarray results published by O’Ryan et al., are included as they were checked by qRT-PCR by Orellana et al.

**TABLE 3:** Biomarkers hypermethylated in *H. pylori* infected children in at least one study included in this review.

**Biomarker**	**No. of studies with significant hypermethylation (references)**	**Biological function**	**Potential role in carcinogenesis**
CALCA	1 (Shin et al., 2011)	Extracellular secreted protein	Controversial
CDH1	1 (Shin et al., 2011)	Cell adhesion protein	Anti-oncogenic
CRABP1	1 (Shin et al., 2011)	Receptor	Controversial
DAPK1	1 (Shin et al., 2011)	Transporter or enzyme	Anti-oncogenic
GATA-4	1 (Alvarez et al., 2013a)	Transcription factor	Anti-oncogenic
GATA-5	1 (Alvarez et al., 2018)	Transcription factor	Anti-oncogenic
GRIN2B	1 (Shin et al., 2011)	Receptor	Anti-oncogenic
THBS1	1 (Alvarez et al., 2013a)	Extracellular secreted protein	Controversial
TIMP3	1 (Shin et al., 2011)	Extracellular secreted protein	Anti-oncogenic
TWIST1	1 (Shin et al., 2011)	Transcription factor	Controversial

*No.: Number.*

### Biomarkers Classified by Biological Function

#### Transcription Factors

Six transcription factors have been identified as differentially expressed or methylated in *H. pylori* infected children at the protein or gene level, three of which have been reported to be overexpressed (p53, β-catenin and C-MYC) and three hypermethylated (TWIST-1, GATA-4, and GATA-5).

p53 is a human tumor suppressor gene, whose expression is constitutively low in healthy tissue and increases in response to DNA damage or other types of cellular stress (Bieging et al., 2014). Inactivation and accumulation of p53 is a hallmark of carcinogenesis, including GC, in which higher p53 levels in malignant tissue are associated with poor prognosis (Yildirim et al., 2015; Zhou et al., 2016). *H. pylori* induction of p53 expression has been described in chronic gastritis and preneoplasic lesions (such as intestinal, metaplasia and dysplasia in adults), especially those associated with CagA-positive strains (Teh et al., 2002; Li et al., 2016). Pediatric studies assessed in this review showed contradictory results, with p53 overexpressed in gastric tissue of *H. pylori* infected children, and directly correlated with gastritis, the magnitude of inflammatory cell infiltration and *H. pylori* tissue density in two studies (Ozturk et al., 2005; Saf et al., 2015); while on the other hand two other studies showed similar expression levels in infected and non-infected children (Nardone et al., 2001; Villarreal-Calderon et al., 2014).

β-catenin acts as a transcriptional regulator in the Wnt signaling pathway (Ebert et al., 2003; Chiurillo, 2015). *H. pylori* promotes the Wnt/β-catenin pathway through several mechanisms: by upregulating Wnt/β-catenin activators, c-Met and EGFR, by downregulating the Wnt/β-catenin suppressors, TFF1 and RUNX3, and also by recruiting tumor-associated macrophages (Song et al., 2015). Villarreal – Calderón et al., describe high membranous expression of β-catenin in gastric biopsies of *H. pylori* infected children as compared to non-infected controls (Villarreal-Calderon et al., 2014).

C-MYC belongs to Myc family of oncogenes (derived from myelocytomatosis viral oncogene). C-MYC is overexpressed in GC compared to normal tissue, and its expression levels are correlated with metastasis and lower survival rates (Zhang et al., 2010; Khaleghian et al., 2015). *H. pylori* gastritis in adults is associated with overexpression of C-MYC (Nardone et al., 1999), and according to Nardone et al. (2001) is also overexpressed in the gastric tissue of infected children, regardless of CagA status, and is associated with high cell proliferation.

TWIST-1 (coding twist-related protein 1) is involved in epithelial-mesenchymal transition and cell-migration processes, and is overexpressed in several human cancers, including GC (Yang et al., 2007; Izadpanah et al., 2017). However, TWIST-1 is hypermethylated in GC and other neoplasms (Gort et al., 2008; Kang et al., 2008; Sakamoto et al., 2015), so its functional significance remains unknown. Hypermethylation has been previously described in gastric mucosa of *H. pylori* infected children and adults (Shin et al., 2011).

GATA-4 and 5 belongs to the GATA family of transcription factors (name derived from DNA consensus sequence to which it binds). Both are cancer suppressor genes involved in cell-differentiation, and the silencing of both GATA-4 and GATA-5 by hypermethyation has been identified in several tumors, including GC (Akiyama et al., 2003; Wen et al., 2010). Furthermore, GATA-4 hypermethylation has been described in intraepithelial neoplasia, gastric dysplasia and *H. pylori* infected tissue in adults (Wen et al., 2010). Alvarez et al. (2013a) demonstrated GATA-4 hypermethylation in pediatric and adult *H. pylori-*induced gastritis, correlating with low expression levels compared to unmethylated samples, however, in GC samples its downregulation was not related to methylation levels. Later, the same group showed GATA-5 hypermethylation in gastric tissue of children and adults infected by *H. pylori*, however, mRNA expression tended to be higher in unmethylated infected tissue compared to non-infected samples, postulating that an initial protective effect of the mucosa in response to infection. Hypermethylation and downregulation was consistently shown in GC samples (Alvarez et al., 2018).

#### Transporters and Enzymes

Four biomarkers identified in this review are classified as transporters or enzymes, two of which have been reported as overexpressed (Bcl-2, COX2), one underexpressed (SLC5A8) and one as hypermethylated (DAPK1).

Bcl-2 (B-cell lymphoma-2) belongs to the intrinsic apoptotic pathway, and *H. pylori* infection is associated with over expression of both the Bcl-2 (which codifies the main anti-apoptotic protein) and Bax genes (pro-apoptotic), with Bax expression predominating in chronic gastritis and Bcl-2 expression in GC. This suggests that disturbances in the balance between Bax and Bcl-2 contribute to *H. pylori* induced carcinogenesis (Bartchewsky et al., 2010). In GC, Bcl-2 is overexpressed, especially in *H. pylori*- associated adenocarcinoma, however, while several studies have assessed the relationship between Bcl-2 expression and prognosis in GC, results have been inconsistent. Additionally, according to a recent meta-analysis, Bcl-2 could be a favorable prognostic marker in Asian populations (Zhang et al., 2004; Cheng et al., 2015; Zhou et al., 2015). These results are in agreement with other studies showing that Bcl-2 not only inhibits apoptosis, but also suppresses cellular proliferative activity (Bélanger et al., 2005). According to Ryszczuk et al. (2016) Bcl-2 is over expressed in antral gastritis of *H. pylori* infected children, in both surface epithelial and gland cells.

COX-2 (Cyclooxygenase 2) is an enzyme which catalyzes prostaglandin synthesis (Vane et al., 1998). It is over expressed in *H. pylori*- infected gastric epithelium, and this over expression is correlated with the presence of severe metaplasia, atrophy and dysplasia (Badary et al., 2017). Expression of the COX-2 gene is significantly increased in human gastric adenocarcinoma tissue as compared to paired gastric mucosal specimens devoid of cancer cells; potential mechanisms for carcinogenesis are apoptosis inhibition, maintenance of cell proliferation and stimulation of angiogenesis in cancer cells (Nuñez et al., 2011). Over expression of COX-2 protein in *H. pylori* infected children, mainly in monocytic cells and myofibroblasts of gastric tissue, was detected and positively correlated with acute and chronic gastric inflammation in one study (Kim et al., 2004). Later, Haruna et al. (2008) showed higher levels of COX-2 mRNA in gastric tissue of infected compared to non-infected children; difference although were not statistically significant, and expression levels were not associated to presence of ulcers.

SLC5A8 (solute carrier family 5 member 8) is a short chain fatty acid transporter that is underexpressed in several human tumors, including GC (Ueno et al., 2004; Ganapathy et al., 2008; Brim et al., 2011). *H. pylori* infection is associated with SLC5A8 underexpression in gastric tissue in symptomatic children and in serum samples in asymptomatic children (Orellana et al., 2016), but expression levels in cancer free infected adults have not been explored.

DAPK1 (death associated protein kinase 1) is a kinase protein with pro-apoptotic function in normal tissue; it is described to be hypermethylated in GC tissue compared to normal tissue, which is associated with poor survival (Yao et al., 2012). Hypermethylation was also reported in gastric tissue of *H. pylori* infected children (Shin et al., 2011), however, methylation levels were higher in infected adults, suggesting an age-related methylation effect.

#### Extracellular Secreted Proteins

Fifteen extracellular secreted proteins have been described as possible biomarkers in *H. pylori* infected children, ten of which were overexpressed (CCL18, CXCL9, CXCL10, CXCL11, CXCL13, LCN2, REG3A, Gastrin, PGI, and PGII), two underexpressed (MUC5AC, TIMP1), and three hypermethylated (CALCA, THBS1, and TIMP3).

CCL18 (C-C Motif Chemokine Ligand 18) is a chemokine involved in migration and activation of leucocytes; it is overexpressed in several cancer tissues, including GC (Schutyser et al., 2005), and is associated with tumor metastasis, as recently reviewed (Huang et al., 2018). It promotes activation of the ERK/NFKB signaling pathway (Hou et al., 2016), suggesting an oncogenic role, which differs from previous reports (Leung et al., 2004). CXCL9, CXCL10, and CXCL11 (C-X-C motif chemokine ligand 9, 10, and 11, respectively) are chemokines that share functions as ligands of CCR3, stimulating antitumoral immunity, but they can also promote proliferation and metastasis in cancer cells in an autocrine pathway, as recently reviewed (Tokunaga et al., 2018). *In vitro* data show that CXCL9, 10, 11/CCR3 axis promote tumor immune-evasion by stimulating expression of the programmed death-ligand 1 (PDL1) in GC cells (Zhang et al., 2018). Overexpression of CXCL9 and 10 is found in GC tissue compared to normal gastric tissue (Raja et al., 2017), however, data on CXCL11 expression in GC was not found. CXCL13 (C-X-C motif chemokine ligand 13) is related to cell proliferation and associated with poor prognosis in several neoplasms, including GC (Fan et al., 2017; Wei et al., 2018). Obayashi et al. (2016) showed CXCL13 overexpression primarily around lymphoid follicles in gastric biopsies from infected children; while Ikuse et al. (2012) showed overexpression of CXCL9, 10, 11, 13, and CCL18 in gastric tissue of infected children, all of whom had histological inflammation and nodular gastritis.

LCN2 (lipocalin 2) is a secreted glycoprotein involved in innate immune response, and its role in GC is controversial; in adults it is overexpressed in gastritis mucosa infected with *H. pylori*, but not in mucosa with intestinal metaplasia, dysplasia or GC (Alpízar-Alpízar et al., 2009). Wang et al. (2010) described that LCN2 overexpression in GC is associated with low survival. Obayashi et al. (2016) postulated that LCN2 overexpression due to *H. pylori* infection in children, demonstrated both in epithelial cells and inflammatory cells in gastric epithelium, may interfere with iron uptake and proliferation of the bacterium, thereby protecting infected gastric mucosa from carcinogenesis.

REG (Regenerating islet-derived) proteins have an anti-apoptotic role in gastric tissue (Parikh et al., 2012), and their overexpression in *H. pylori* infected gastric mucosa is associated with neutrophil activity and chronic inflammation (Yoshino et al., 2005). REG3A, a member of this family, is overexpressed in GC, and promotes the proliferation, migration, invasion and adhesion of GC cells by regulating the JAK2/STAT3 signal pathway (Chen et al., 2017). However, previous reports showed under expression of REG3A in primary GC and GC cell lines (Choi et al., 2007). Obayashi et al. (2016) showed overexpression of REG3A in inflammatory cells in lamina propia, but not epithelial cells of gastric epithelium, in both infected children and adults, which reflects a role in chronic gastritis.

MUC5AC (mucin 5AC) is a secreted mucin underexpressed in epithelial neoplasms (Corfield et al., 2001). A recent meta-analysis describes that *H. pylori* infection is associated with lower MUC5AC expression in the gastric epithelium compared to non-infected patients (Niv, 2015). Furthermore, MUC5AC is underexpressed in GC tissue compared to healthy tissue, and its decreased expression is associated with more aggressive tumor behavior (Javanbakht et al., 2017). However, MUC5AC expression in other tissues, such as lung and pancreas, induces cell proliferation and migration, acting as a pro-oncogenic factor (Yamazoe et al., 2010; Lakshmanan et al., 2016). In children, its expression is lower in *H. pylori* infected mucosa compared to non-infected samples, specifically in children >5 years of age (Park et al., 2015).

Matrix metalloproteinase (MMPs) are involved in tumor invasion, and their activity is regulated by inhibitors such as tissue inhibitor metalloproteinase-1 (TIMP1) (Kessenbrock et al., 2010). However, pro-oncogenic functions of TIMP proteins have been described (Jackson et al., 2017). TIMP1 expression is up regulated in GC, with tumor-associated myofibroblasts acting as the main source of increased TIMP1 expression locally (Alpízar-Alpízar et al., 2016). Also, elevated protein levels in tumor tissue and plasma from GC patients are associated with poor outcomes (Grunnet et al., 2013). However, as reviewed here, *H. pylori* infection in children is associated with decreased serum expression of TIMP1 (Rautelin et al., 2010), which conflicts with previously exposed data in adults and requires further study.

In contrast to TIMP1, evidence suggests that TIMP3 has an anti-oncogenic function (Anand-Apte et al., 1996; Ahonen et al., 1998). Increased methylation and lower expression levels have been shown in GC compared to normal gastric tissue; a direct relationship between methylation levels, lymph node metastasis and more advanced cancers has also been described (Yao et al., 2012; Guan et al., 2013). The hypermethylation of TIMP3 in infected children (Shin et al., 2011) may reflect the beginning of a downregulation process.

CALCA (calcitonin related polypeptide alpha), codifies procalcitonin and procalcitonin-related peptides, precursors of calcitonin, a regulator of calcium homeostasis. It is over expressed in lung and medullar thyroid carcinomas, also playing a relevant role in T-cell and B-cell regulation in these malignancies (El Hage et al., 2008). CALCA hypermethylation has been described in several hematological and solid tumors, including GC, and is associated with a poor prognosis (Paixão et al., 2006; Yao et al., 2012; Martinelli et al., 2016). Gastric hypermethylation of CALCA has been described in *H. pylori* infected children (Shin et al., 2011).

THBS1 (Thrombospondin 1) is a matrix glycoprotein with controversial role in cancer, with some reports showing an antioncogenic function, and others a pro-oncogenic role (Grossfeld et al., 1997; Maeda et al., 2001; Kazerounian et al., 2008). Specifically in GC, decreased THBS1 expression has been associated with a poor prognosis (Eto et al., 2015), but higher expression in cancer tissue has been correlated with cell invasion and migration (Huang et al., 2017). Alvarez et al. (2013a) describe hypermethylation of THBS1 in gastric tissue of infected adults and children. Expression level in unmethylated samples was higher in infected children, however, comparison of expression level in total samples between infected and not infected children is not shown. In gastric cancer tissue, meanwhile, its downregulation was not associated with methylation levels (Alvarez et al., 2013a). Thus, the role of THBS1 in carcinogenesis is controversial, and its hypermethylation in *H. pylori* infected children (Alvarez et al., 2013a) may or may not be related to future carcinogenesis in gastric tissue.

Pepsinogens (PGs) I and II are precursors of pepsin, produced in gastric cells, and in case of PGII, also by duodenal cells (Samloff, 1971; Samloff and Liebman, 1973). Because they are secreted to the gastric lumen, PGI and II are currently used as serum biomarkers of the functional and anatomical integrity of gastric mucosa (Shiota and Yamaoka, 2014). In *H. pylori*-induced gastritis, both PGI and II are upregulated, with a greater increase in PGII and a consequent decrease in the PGI/II ratio (Kim and Jung, 2010). Changes in PG expression during infection are caused by gastric inflammation and by direct stimulation of *H. pylori* lipopolysaccharides on gastric cells, and are reversed after eradication treatment (Young et al., 2002). A decrease in PGI, PGII and the PGI/II ratio occurs in atrophic gastritis and GC, as extensively reviewed (Huang et al., 2015). In children, expression of PGI and II in gastric tissue of infected children has been reported to increase at the protein level (Roma et al., 2006) and decrease at the mRNA level (Ikuse et al., 2012). However, various studies have shown a consistent increase in serum PGII in both symptomatic (Lopes et al., 2006; De Angelis et al., 2007; Koivusalo et al., 2007; Guariso et al., 2009; Kassem et al., 2017) and asymptomatic (Fukuda et al., 2003; Rogers et al., 2003; Nakayama et al., 2017) infected children. In case of PGI, most studies show serum overexpression in both symptomatic and asymptomatic infected children (Fukuda et al., 2003; Rogers et al., 2003; Lopes et al., 2006; Roma et al., 2006; Guariso et al., 2009; Kassem et al., 2017; Nakayama et al., 2017; Kienesberger et al., 2018); but 2 studies reported no difference between infected and non-infected children (De Angelis et al., 2007; Koivusalo et al., 2007). However, De Angelis et al. (2007) reported serum overexpression specifically among infected children older than 10 years, while Koivusalo et al. (2007) included mostly younger children than other studies. Thus, an age-dependant induction in PGI, as reported in cohort studies (Pérez-Pérez et al., 2003) could potentially explain this variability in case-control results.

Gastrin is a peptide hormone produced by gastrointestinal cells, in a process involving several intermediates forms of which Gastrin 17, an intermediate form generated from preprogastrin, is the most abundant circulating form (Smith et al., 2017). It is responsible for initiating gastric acid release in the stomach in response to stimulus. During gastric cancer development, atrophic gastritis and hypergastrinemia have been proposed to be critical factors for both diffuse type and intestinal types of GC (reviewed in Waldum et al., 2017), by stimulating cell proliferation and other mechanisms not completely elucidated (Copps et al., 2009; Smith et al., 2017; Waldum et al., 2017). In children, serum gastrin and gastrin 17 have been reported to be overexpressed in *H. pylori* infected children in five studies included in this review (Kato et al., 2004; Maciorkowska et al., 2006; Plonka et al., 2006; Czaja et al., 2008; Guariso et al., 2009). On the other hand, five studies (Fukuda et al., 2003; Roma et al., 2006; Xie et al., 2006; De Angelis et al., 2007; Koivusalo et al., 2007) showed no differences in gastrin or gastrin 17 serum or gastric levels according to infectious status. Most studies [with the exception of Plonka et al. (2006) and Fukuda et al. (2003)] were performed in symptomatic children. One study (Lopes et al., 2006) showed lower gastrin levels among infected symptomatic children compared to controls.

#### Receptors

Four biomarkers highlighted here have a primary biological function as receptors: EGFR and PPAR-Y (overexpressed in *H. pylori* infected individuals), CRABP2 (hypermethylated), and GRIN2B (hypermethylated).

EGFR (epidermal growth factor receptor) is involved in cell proliferation signaling (Wee and Wang, 2017). In gastric cell lines and animal models, *H. pylori* induces EGFR expression and activation through phosphorylation; EGFR inhibition downregulates *H. pylori*-induced epithelial inflammatory responses, such as DNA damage and gastric carcinogenesis (Keates et al., 2007; Kim et al., 2013; Sierra et al., 2017). In humans, EGFR is over expressed in GC compared with normal gastric tissue and correlates with poor prognosis (Gao et al., 2013; Petrini et al., 2017). Therefore, anti-EGFR agents have been evaluated for treatment of advanced GC, with variable results between studies, and no overall benefit according to a recent meta-analysis (Kim et al., 2017). In infected children, EGFR is overexpressed mainly in epithelial gastric cells (Maciorkowska et al., 2009; Ryszczuk et al., 2016).

CRABP1 (Cellular retinoic acid-binding protein 1) is responsible for binding retinoic acid transporting it into cells. Evidence for its role in carcinogenesis is controversial, showing both pro-apoptotic and pro-metastatic properties *in vitro* (Kainov et al., 2014; Persaud et al., 2016). Its hypermethylation and/or underexpression has been described in colorectal, breast, ovarian, esophageal and cervical cancers (Tanaka et al., 2007; Miyake et al., 2011; Liu et al., 2015; Arellano-Ortiz et al., 2016; Cha et al., 2016). However, to our knowledge there is no evidence as to the role of its expression or methylation status in GC, making the finding of its *H. pylori*-mediated hypermethylation in children, described by Shin et al. (2011) unclear.

GRIN2B (Glutamate Ionotropic Receptor NMDA Type Subunit 2B) codifies for a glutamate receptor expressed on the surface of cancer cells (Deutsch et al., 2014), and its hypermethylation and silencing have been reported in several solid tumors, including GC (Kim et al., 2006; Liu et al., 2007; Kang et al., 2008; Tamura et al., 2011). In GC, *H. pylori* infection showed no significant correlation with GRIN2B hypermethylation (Kang et al., 2008). Shin et al. (2011) described the hypermethylation of both CRABP1 and GRIN2B in gastric tissue of *H. pylori* infected children compared to non-infected controls. Furthermore, samples from infected adults showed higher methylation than infected children (Shin et al., 2011), suggesting an age-related methylation effect in long-term infections, which may be related to future carcinogenesis.

PPAR-Υ (Peroxisome proliferator-activated receptor gamma) is a nuclear hormone receptor, which also acts as a transcription factor regulating expression of genes involved in metabolism and cell proliferation. Depending on the cell context, it can act as antiproliferative and antitumorigenic, or antiapoptotic and oncogenic (Vella et al., 2017). In gastric tissue, overexpression has been described in GC and dysplasia compared to gastritis (Yao et al., 2010), but treatment with PPAR-Υ agonist inhibits cell proliferation (Sato et al., 2000). Other studies have described downregulation in GC and association with survival (Yu and Xin, 2013). In children, overexpression of PPAR-Υ mRNA has been described in gastric tissue of infected children compared to non-infected controls (Haruna et al., 2008), but because its role in GC is not clear, the relative importance of its overexpression in children requires further analysis.

#### Signaling Proteins or Kinases

p21 is classically defined as a tumor suppressor protein, however, it is generally overexpressed in cancer tissue, and there is increasing evidence that p21 plays an oncogenic role (Georgakilas et al., 2017). In GC tissue, p21 is overexpressed and its expression has also been associated with poor prognosis (Luo et al., 2014; Altun et al., 2015). *H. pylori* infection in *in vitro* models are correlated with p21 overexpression (Xia et al., 2008); in the pediatric population Saf et al. (2015) described the overexpression of p21 in gastric tissue of *H. pylori* infected children, however, in a study published before the date range stated in our search strategy (not included in tables) gastric expression was comparable to non-infected children (Jones et al., 1997), showing contradictory results in children.

PIM2 (proviral integration site of Moloney virus) is a kinase with anti-apoptotic function *in vitro* (Blanco-Aparicio and Carnero, 2013), and its overexpression has been described mainly in hematologic neoplasms, such as multiple myeloma, as well as in solid tumors, such as colorectal and liver carcinomas (Gong et al., 2009; Zhang et al., 2015; Ramachandran et al., 2016). Recently, PIM2 overexpression associated with poor prognosis has been described in GC, related with suppression of reactive oxygen species and endoplasmic reticulum stress mediated apoptosis (Xin et al., 2018). In children, PIM2 is overexpressed in gastric epithelium of *H. pylori* infected children (Obayashi et al., 2016).

#### Cell-Adhesion Proteins

CDH1 (cadherin 1) has cell adhesion as a primary function and hypermethylation has been described in both GC (Zazula et al., 2006) and in relation to *H. pylori* infection; furthermore, its hypermethylation is reversed following eradication therapy (Choi et al., 2016). Shin et al. (2011) describe *H. pylori*-associated hypermethylation in gastric tissue of children, at levels similar to adults.

VCAM-1 (Vascular Cell Adhesion Molecule 1) overexpression is related to angiogenesis and metastasis in cancer, including GC (Kong et al., 2018); in which both local expression in gastric tissue and serum levels are directly associated with poor prognosis (Velikova et al., 1997; Ding et al., 2003). In children, Maciorkowska et al., described that serum concentrations of VCAM-1 were higher in symptomatic children with *H. pylori-*associated gastritis compared to non-infected children; they also compared children with positive IgG against *H. pylori* but with no active gastric infection (Maciorkowska et al., 2005), showing that serum levels of VCAM-1 correlate with *H. pylori*-induced gastric inflammation and damage.

DC-SIGN (Dendritic Cell-Specific Intercellular adhesion molecule-3-Grabbing Non-integrin) is a lectin that can act as a pattern recognition receptor, recognizing carbohydrates from microorganisms, for initiation of the immune response. In addition, it can mediate cell migration and adhesion of dendritic cells, and in cancer, it can mediate immune escape of tumor cells. Expression of DC-SIGN is normally low in gastric tissue, being overexpressed in gastric cancer (Domínguez-Soto et al., 2011), where it promotes proliferation, cell cycle progression, migration and invasion of GC cells *in vitro* (Li et al., 2020). In children, DC-SIGN is overexpressed in gastric tissue of *H. pylori* infected children, which has been directly correlated with the magnitude of inflammation (Wu et al., 2014); this correlates with *in vitro* results, showing that *H. pylori* induces DC-SIGN expression in gastric epithelial cells and a Th1 immune response (Wu et al., 2014). Although this reflects epithelial immune response against the pathogen, its relevance in future carcinogenesis needs to be elucidated.

#### Cell Proliferation Markers

Ki-67 is widely used as a cell proliferation marker (Li et al., 2015), but its function in carcinogenesis is not clear. Ki-67 is overexpressed in intestinal metaplasia and GC tissue compared to healthy tissue, but has not shown a clear prognostic value in GC (Zheng et al., 2010; Böger et al., 2016). Its expression is also increased in *H. pylori* infected mucosa in adults (Sagheb et al., 2016), with concordant results in all pediatric studies reviewed here. Overexpression of Ki67 in gastric tissue of infected children is indicative of proliferative activity, extending to surface epithelium, which is not observed in non-infected controls (Muñoz et al., 2007). Gastric Ki67 expression in infected children is positively correlated with acute and chronic inflammatory infiltrating cells (Kim et al., 2004; Ozturk et al., 2005; Muñoz et al., 2007), intestinal metaplasia and glandular atrophy (Kim et al., 2004) and *H. pylori* density (Kim et al., 2004; Ozturk et al., 2005; Saf et al., 2015).

#### Micro RNAs

Two microRNAs, involved in post-transcriptional gene expression regulation are significantly overexpressed in gastric tissue of symptomatic infected children. miRNA-146a and miRNA-155 have been described as downregulated in GC (Hou et al., 2012; Li et al., 2012), but also as a potential oncogenes *in vitro* (Shomali et al., 2017; Qu et al., 2018). Cortés-Márquez et al. (2018) described higher gastric expression in both miRNAs in children, especially in those with severe gastritis, as observed in adults and in animal models. Because their role in carcinogenesis is controversial, further studies are needed to determine the potential role of overexpression among infected children.

### Biomarkers Identified in Symptomatic Versus Asymptomatic Infected Children

Only 4/37 biomarkers identified in this review were assessed in asymptomatically infected children (SLC5A8, PGI, PGII, and gastrin); all four were evaluated in blood or serum samples, as upper endoscopies are not performed in this group. SLC5A8, PGI, and PGII were also evaluated in gastric tissue of symptomatic children, showing comparable results (except for PGI and PGII mRNA expression in gastric tissue, as commented before). These finding suggest that gastric damage can occurs among infected children not only when symptoms become evident, but also in apparently healthy children. For gastrin, reported results seem controversial as expression in gastric tissue of symptomatic children was increased (Lopes et al., 2006) while serum levels were decreased in asymptomatic children (Plonka et al., 2006).

## Discussion

In this extensive review we identified 37 molecules, previously detected in adults with cancer, differentially expressed and/or methylated, in gastric tissue and/or blood/serum of *H. pylori* infected children, when compared to non-infected controls. Based on our review, we propose that 13 of these biomarkers require special attention, due to the concordance between expression and/or methylation status in infected children, data in adults (with *H. pylori*-induced gastritis and gastric cancer) and their consistent role in carcinogenesis (Tables 1–3): β-catenin, C-MYC, GATA-4, DAPK1, CXCL13, DC-SIGN, TIMP3, EGFR, GRIN2B, PIM2, SLC5A8, CDH1, and VCAM-1. Six of them (β-catenin, C-MYC, CXCL13, DC-SIGN, EGFR, and PIM2) are consistently described as oncogenes according to the literature, and are overexpressed at the protein or mRNA level in gastric tissue among infected children and gastric cancer patients. VCAM-1, a molecule described as an oncogene in adults, is overexpressed in serum of children with *H. pylori*-induced gastritis, representing a potential non-invasive biomarker of *H. pylori-*induced damage in both symptomatic and asymptomatic children. Five molecules (DAPK1, TIMP3, GRIN2B, SLC5A8, and CDH1) are tumor suppressor genes consistently downregulated and/or hypermethylated in GC and in *H. pylori* infected children. The fact that genes or proteins with a known oncogenic or antioncogenic function have a consistent dysregulation in *H. pylori* infected children, suggests a pathogenic role of infection during early stages, and supports the evaluation of treatment and/or follow-up strategies for infected children, even in absence of symptoms, due to their potential long-term consequences.

Pepsinogen I and II have no described oncogenic effect at the cellular or molecular level, but they reflect integrity of gastric mucosa; overexpression at the protein level in serum of infected children, consistent with data of *H. pylori* induced gastritis in adults, suggests that these biomarkers can be useful for determining gastric damage or inflammation induced by *H. pylori* in children. However, according to studies reviewed here, even though elevated serum pepsinogens levels predicts *H. pylori* infection (De Angelis et al., 2007; Guariso et al., 2009), they have not always correlated with histological gastritis (Lopes et al., 2006). The use of these proteins as seric biomarkers of gastric damage in asymptomatically infected children should be prospectively studied, as the levels are age-dependant.

Overexpression of gastrin is an important factor in the development of gastric cancer as discussed here. Despite the lack of consensus in studies reviewed here on the role of overexpression among infected compared to non-infected children, prospective and long term studies are needed to determine if persistent infection produces sustained hypergastrinemia and possible carcinogenesis as children progress toward older ages.

The overexpression of Ki67 in infected children, a proliferation marker with an unknown function in carcinogenesis, may represent an early biomarker of gastric carcinogenesis, but may also only reflect high levels of cell proliferation during an inflammatory process, however, this could also be considered a reflection of the pathogenesis of *H. pylori* infection since childhood, and its implication on future pathogenesis, together with PGI, PGII and gastrin should be explored further.

p53 has a well known antioncogenic function however, its overexpression has been demonstrated in gastric cancer during preneoplasic stages, and also in *H. pylori*-infected children as commented before, though there is no consensus in studies reviewed here. While it remains unclear whether this accumulated protein is inactivated, it may constitute a valuable early biomarker of bacteria-induced, chronic gastric inflammation and potential carcinogenesis.

Another group of biomarkers identified in this review have contradictory functions in cancer development, however, they are consistently overexpressed (COX2, CCL18, CXCL9, CXCL10, CXCL11), underexpressed (MUC5AC) or hypermethylated (TWIST-1) in *H. pylori*-infected children and adults with *H. pylori*-induced gastritis and/or GC. This suggests that despite an unclear role in carcinogenesis, they are associated with gastric damage and GC in adulthood, and their demonstrated dysregulation in infected children should be prospectively explored, as they could represent either transitory molecular changes associated with infection and inflammatory response and/or early changes in the natural history of gastric damage during long-term infections.

Lastly, a group of molecules differentially expressed in *H. pylori* infected children lack sufficient evidence as to either their role in gastric damage in adults with *H. pylori*- induced gastritis or GC (BCL-2, LCN2, REG3A, CALCA, THBS1, CRABP1, PPAR-Y) or dysregulation in adulthood is contradictory with findings in children (miRNA146a, miRNA155, TIMP1). Additionally, in the case of p21, there is disagreement in its expression levels in *H. pylori* infected children. Taken together, this group of molecules and their expression over time require more in depth studies in order to include them as potential biomarkers of early oncogenic gastric damage in *H. pylori* infected children.

The fact that various genes and proteins involved in GC are significantly underexpressed, overexpressed or hypermethylated in *H. pylori* infected children underscores the possibility that chronic infection in children, even if asymptomatic, is causing the development of a progressive malignant disease in a subset of children to be manifested later in life. However, understanding the dynamics and natural history of *H. pylori* infection in children remains to be fully elucidated. As recently reviewed, most of the epidemiological studies on *H. pylori* infection in children are cross-sectional, thus lacking information on persistence or spontaneous clearance of the infection (Zabala Torrres et al., 2017). Importantly, the main limitation of this review lies in that all the included studies have a cross-sectional design, some of them with low number of subjects; thus whether changes in gene expression generated by *H. pylori* infection endure in persistently infected children, and how these changes impact clinical outcomes needs to be explored further. A few studies have assessed how eradication therapy affects gene expression in *H. pylori* infected adults, showing a significant decrease in p53, MDM2 (Multiple double minute 2), C-MYC, Ki-67 and cyclin D1 expression in gastric tissue following eradication therapy (Kodama et al., 2005; Alshenawy and Alshafey, 2009; Triantafyllou et al., 2016). However, to our knowledge there are no published studies evaluating gene expression both pre- and post-eradication therapy in children.

## Conclusion

**FIGURE 3:**
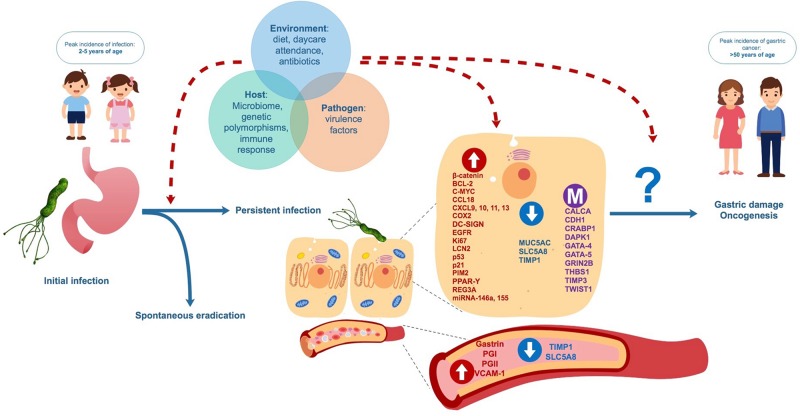
Proposed natural evolution of *H. pylori* infection and biomolecular changes from asymptomatic childhood infection to adult gastric diseases. Interaction between factors related to the environment, the host and the pathogen may influence persistence *H. pylori* of infection, biomolecular changes in gastric cells and blood and the future development of benign and malign gastric disease.

We have summarized and discussed a number of studies indicating that early *H. pylori* infection during childhood may generate differences in the expression and methylation of genes potentially involved in carcinogenesis, which could be affecting the risk of gastric damage or even future GC development. At the moment it is not possible to assure that modified expression levels and/or methylation could be used as biomarkers for a predisposition to GC in adulthood. The role of many of these biomarkers in carcinogenesis is controversial, involving broad biological functions related to immune response, cell cycle, and apoptosis. Also, in case of biomarkers studied in gastric tissue, site of sampling (antrum vs. corpus) may differ between adult and child patients. These specific roles may be altered in both oncogenic processes and chronic inflammation, as they are closely related to gastric damage pathogenesis (Kalisperati et al., 2017). Several factors not detailed in this review, including environmental stimulus, gastric and/or intestinal microbiome profiles, bacterial virulence factors and host genetic polymorphisms in genes related to innate or adaptive immunity may influence not only the risk of *H. pylori* persistence in childhood, but probably also whether biomolecular changes determine gastric damage and/or future carcinogenesis (Figure 3; Haley and Gaddy, 2015; Alarcón et al., 2017). Long-term observational studies on infected and non-infected children are necessary to assess the spontaneous evolution of gene expression and methylation, and the response to eradication therapy. Furthermore, more in-depth screening of these molecules is required, in order to clarify their role in the development of chronic infection and in future outcomes of infection. In the meantime, this review should aid researchers in selecting the most suitable targets in the quest for potentially useful biomarkers of future GC risk in *H. pylori* infected children.

## Author Contributions

SG and MO’R conceived the original idea. SG performed literature search and study selection. SG and MO’R wrote the manuscript with substantial support from YL, JT, and AL. All authors approved the final manuscript.

## Conflict of Interest

The authors declare that the research was conducted in the absence of any commercial or financial relationships that could be construed as a potential conflict of interest.
