# Supplementary figures and images for: Gastric Damage and Cancer-Associated Biomarkers in Helicobacter pylori-Infected Children
Source: Front Microbiol. 2020 Feb 12;11:90. doi: 10.3389/fmicb.2020.00090 (PMC7029740; doi:10.3389/fmicb.2020.00090)

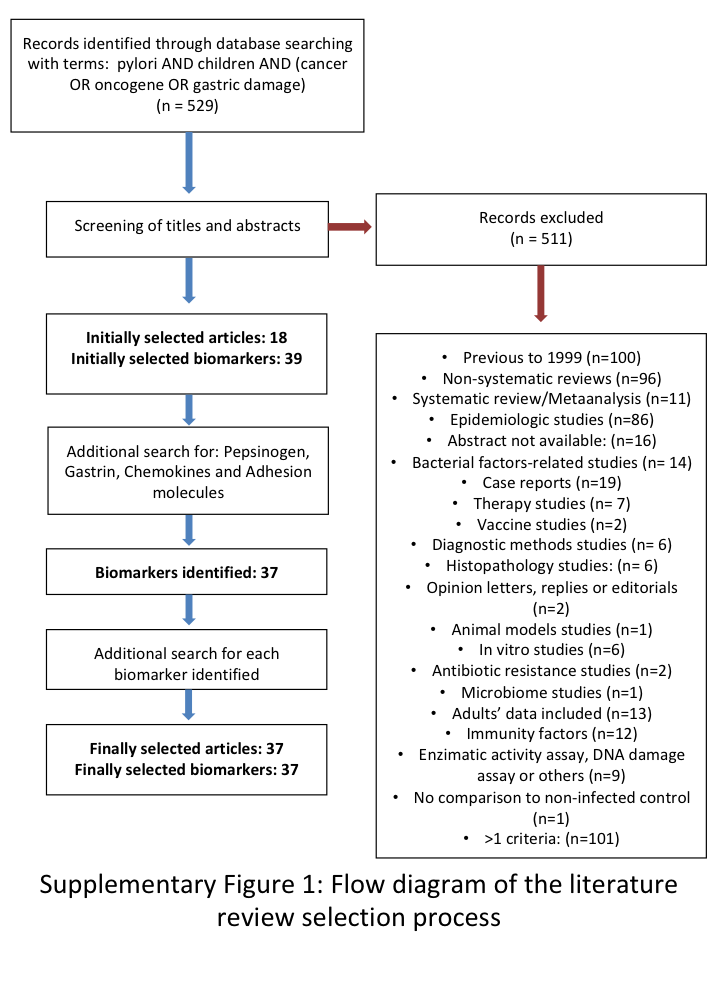

Supplement: Supplementary file 1 [file Image_1.tiff]
