# Supplementary material for: Gastric Damage and Cancer-Associated Biomarkers in Helicobacter pylori-Infected Children
Source: Front Microbiol. 2020 Feb 12;11:90. doi: 10.3389/fmicb.2020.00090 (PMC7029740; doi:10.3389/fmicb.2020.00090)
Supplement: Supplementary file 2 [file Table_1.docx]

**Supplementary Table 1: Published studies on biomarkers expression levels included in this review**

| **Reference (Country)** | **N (children)** | **Age (range in years)*** | **Gender**  **(% female)** | **Abdominal Symptoms** | **HP status assessment** | **Expression assessment method / samples** | **No. of differentially expressed biomarkers according to infection status (details)** | **No. of biomarkers not differentially expressed according to infection status (details)** | **Comments** |
| --- | --- | --- | --- | --- | --- | --- | --- | --- | --- |
| Nardone et al., 2001 (Italy) | **53**  26 (+)  27 (-) | 3-15 | (+): 42%  (-): 44% | Yes | Urea breath test, histopathology and immunohistochemistry | Immunohistochemistry / gastric biopsy | 1  (C-MYC) | 1  (p53) | - |
| Fukuda et al., 2003 (Japan) | **300**  **37 (+)**  **263 (-)** | 8.0 ± 4.9 | 42% | No | Serology (serum IgG anti H. pylori and anti CagA) | RIA / serum | 2  (Pepsinogen I, Pepsinogen II) | 1  (Gastrin) | - |
| Rogers et al., 2003 (Guatemala) | **172**  **144 (+)**  **28 (-)** | (+):10.9 ± 1.1  (-):10.4 ± 1.4 | NA | Yes* | UBT | Gastrin: double antibody immu- noassay/ serum  PG: RIA/ Serum | 2  (Pepsinogen I, Pepsinogen II) | 0 | Both symptomatic and asymptomatic children included. |
| Kato et al., 2004 (Japan) | **15**  **10 (+)**  **5 (-)** | 10-17 | NA | Yes | Histopathology, Urease test and UBT (>1) OR culture of gastric biopsy | RIA / Serum | 1  (Gastrin) | 0 | Gastrin: measured at basal and postprandial levels. |
| Kim et al., 2004 (Korea) | **65**  52 (+)  13 (-) | (+): 3-17  (-): 6-13 | (+): 51%  (-): 61% | Yes | Urease test and histopathology | Immunohistochemistry / gastric biopsy | 2  (COX-2, Ki67) | 0 | - |
| Maciorkowska et al., 2005 (Poland) | **106**  59 (+)  47 (-) | (+): 2-19  (-): 3-19 | (+): 50%  (-): 55% | Yes | Histopathology and serology | ELISA / serum | 1  (VCAM-1) | 2  (ICAM-1, P-selectin) | Patients were divided in 3 groups: I: *H. pylori* gastritis with positive serology (shown as positive in this table). II: seropositive with no current infection, and III: seronegative. VCAM-1 was overexpressed in group I compared to II and III. |
| Ozturk et al., 2005 (Turkey) | **54**  35 (+)  19 (-) | 4-18 | 62% | Yes | Histopathology | Immunohistochemistry / gastric biopsy | 2  (p53, Ki67) | 0 | - |
| Lopes et al., 2006 (Portugal) | **92**  41 (+)  51 (-) | (+): 3.7-17  (-): 2.1-15.5 | 52.2% | Yes | Urease test, culture of gastric sample and histopathology | RIA / serum | 3  (Pepsinogen I, Pepsinogen II, Gastrin) | 0 |  |
| Maciorkowska et al., 2006. (Poland) | **106**  **88 (+)**  **18 (-)** | (+): 2-18  (-): 5-17 | (+): 48%  (-): 66.7% | Yes | Serology (with or without Histopathology) | RIA / serum | 1  (Gastrin) | 0 | Patients sero-positive for *H. pylori* were divided in two groups: with or without gastritis. Seropositive children with and without *H. pylori*-gastritis had higher gastrin levels compare to non-infected. |
| Plonka et al., 2006 (Poland) | **146**  53 (+)  93 (-) | 6-17 | 60% | No^$^ | UBT | RIA / serum | 1  (Gastrin) | 0 | Ghrelin, leptin and TNFalpha levels were also measured, but were not considered according to focus of this review.  ^$^Dyspeptic symptoms were found in 25 children; % of symptomatic children in each group is NA |
| Roma et al., 2006 (Greece) | **101**  45 (+)  56 (-) | (+): 4-16  (-): 4.5-16 | 51% | Yes | Culture of gastric sample, or histopathology and rapid urease test | RIA / serum | 1 (Pepsinogen I) | 1 (Gastrin 17) | Non infected children: included Non *H. pylori* gastritis and children with normal mucosa |
| Xie et al., 2006 (China) | **83**  **41 (+)**  **42 (-)** | NA | NA | NA | Rapid urease test and histopathology | Immunohistochemistry / gastric biopsy | 0 | 2  (Gastrin, Somatostatin). | Study made using gastric samples from endoscopy of children. No details about age, gender and indication of endoscopy are provided. |
| De Angelis et al., 2007 (Italy) | **45**  18 (+)  27 (-) | (+): 5-17  (-): 3-16 | (+): 55%  (-): 59% | Yes | Histopathology, Rapid urease test and culture (>1) | ELISA / serum | 1 (Pepsinogen II) | 2 (Pepsinogen I, Gastrin 17) | Pepsinogen I was overexpressed in *H. pylori*-infected children >10 years old, but not in all-age infected group. |
| Koivusalo et al., 2007 (Finland) | **80**  **30 (+)**  **50 (-)** | (+): 2.4-12.1  (-): 0.6-18.2 | 46% | Yes^$^ | Histopathology and rapid urease test with or without positive culture | ELISA / serum | 1 (Pepsinogen II) | 2 (Pepsinogen I, Gastrin 17) | ^$^ 40/80 patients were submitted to endoscopy because an adittional cause: gastroesophagus reflux, suspicious of celiac disease, Chron’s disease, and surveillance of previous gastric or esophagic abnormalities.. |
| Muñoz et al., 2007 (Mexico) | **88**  44 (+)  44 (-) | (+): 2-16  (-): 4-16 | NA | Yes | UBT, culture of gastric samples, and histopathology | Immunohistochemistry / gastric biopsy | 1  (Ki67) | 0 | - |
| Czaja et al., 2008 (Poland) | **184**  **60 (+)**  **124 (-)** | 5.6-18 | 57% | Yes | Urease test | RIA / serum | 1  (Gastrin) | 0 |  |
| Haruna et al., 2008 (Japan) | **24**  12 (+)  12 (-) | (+): 3.2-17.5  (-): 1.2-18.3 | (+): 75%  (-): 33% | Yes ^$^ | Stool antigen test, UBT, histopathology, and culture (>1 positive) | qRT-PCR / gastric biopsy | 2  (COX2, PPAR-ϒ) | 0 | ^$^Children were refered to endoscopy because of abdominal symptoms or anemia. |
| Guariso et al., 2009 (Italy) | **407**  97 (+)  310 (-) | 0,25-18 | NA | Yes | Histopathology and culture | ELISA / serum  RIA/ serum (total gastrin) | 4  (Pepsinogen I, Pepsinogen II, Gastrin 17, total gastrin) | 0 | Data about patients with celiac disease are not detailed.  Age data specifically about *H. pylori* infected and non-infected children without celiac disease is NA. |
| Maciorkowska et al., 2009 (Poland) | **44**  30 (+)  14 (-) | 5-18 | (+): 46%  (-): 50% | Yes | Rapid urease test and histopathology | Immunohistochemistry / gastric biopsy | 1 (EGFR) | 0 | - |
| Rautelin et al., 2010 (Finland) | **60**  26 (+)  34 (-) | (+): 2-13  (-): 0.8 -17 | (+): 50%  (-): 23% | Yes | Culture of gastric sample, or histopathology and rapid urease test | ELISA / serum | 1  (TIMP1) | 6  (MMP-2, -7, -8, -9, HNE, MPO) | - |
| Ikuse et al, 2012 (Japan) | **12**  6 (+)  6 (-) | (+): 10.1-14.6  (-): 10.3-15.5 | (+): 50%  (-): 50% | Yes | Culture of gastric samples and UBT | Microarrays and qRT-PCR / gastric biopsy | Microarrays: 21 in antrum/ 16 in corpus ^◆^  qRT-PCR: 6  (LCN2, CCL18, CXCL9, CXCL11, Pepsinogen I, pepsinogen II, CXCL13^✜^, CXCL10^✜^) | Microarrays: ND**  qRT-PCR: 0 | ^◆^: Only the those whose expression levels were checked by qRT-PCR and/or immunohistochemistry are listed here  ^✜ ^ Statistically significant when analyzed with significance value of p<0.05, but no with p<0.01  **: The total number of genes analyzed in microarrays is not specified |
| Alvarez et al., 2013(a) (Brazil) | **50**  (+) 22  (-) 28 | 8 ± 4 | NA | Yes | Histopathology and PCR of gastric biopsies | qRT-PCR / gastric biopsy | 0 | 2  (HIC1, GATA4, THBS1) | THBS1 expression level in unmethylated samples were higher in infected children; however a comparison of expression level in total samples between infected and non-infected children is not shown. |
| Villarreal-Calderón et al., 2014 (Mexico) | **82**  36 (+)  46 (-) | 8.3±4.8 | 57% | Yes | Histopathology and immunohistochemistry | Immunohistochemistry / gastric biopsy | 1  (β-catenin) | 6  (CDX2, EphB4, MMP3, MIF, p53, E-cadherin) | - |
| Wu et al., 2014 (China) | **72**  42 (+)  30 (-) | 2-17 | 43% | Yes | Histopathology | Immunohistochemistry / gastric biopsy | 1  (DC-SIGN) | 0 | - |
| O’Ryan et al., 2015 (Chile) | **53**  22 (+) persistent  22 (+) transient  9 (-) | 1.5 – 4.8 | NA | No | Stool antigen test | Microarray / whole blood | 10  (CDT1, NUSAP1, TGFα, BTG3, TIAM1, C3ORF38, h-CCPX, HIPK2, IFN- γ, SLC5A8) | ND** | Data about age and gender of 53 children which blood sample was analyzed for microarray is not available  **: In the case of microarray results, the total number of genes analyzed is not specified |
| Park et al., 2015 (Korea) | **168**  83 (+)  85 (-) | 1.4-14.8 | (+): 46%  (-): 51% | Yes | Histopathology | Immunohistochemistry / gastric biopsy | 1  (MUC5AC) | 2  (MUC2, MUC6) | - |
| Saf et al., 2015 (Turkey) | **60**  20 (+)  40 (-) | 3-12 | (+): 40%  (-): 50% | Yes | Stool antigen test, rapid urease test and histopathology | Immunohistochemistry / gastric biopsy | 3  (p53, p21, Ki67) | 0 | Patients were divided in 3 groups: I: No gastritis II: *H. pylori*-negative gastritis, and III: *H. pylori*-positive gastritis. Significant difference in p53 and Ki67: group III vs I  P21: Group III vs I and II |
| Nakayama et al., 2016 (Japan) | **454**  14 (+)  440 (-) | 12-15 | (+): 42%  (-) 50% | No | Serology (plus UBT in a subset of patients) | CLEIA / serum | 2  (Pepsinogen 1, Pepsinogen II) | 0 |  |
| Obayashi et al., 2016 (Japan) | **12**  6 (+)  6 (-) | 4.9-5.6 | (+): 33%  (-): 33% | Yes | Culture of gastric samples and a second test (serology, UBT or stool antigen test) | Microarray, qRT-PCR, immunohistochemistry / gastric biopsy | Microarrays: 27 ^◆^  qRT-PCR and immunohistochemistry: 4  (CXCL13, LCN2, PIM2, REG3A) | Microarrays: ND**  qRT-PCR and immunohistochemistry: 1 (OLFM4) | ^◆^: Only the those whose expression levels were checked by qRT-PCR and/or immunohistochemistry are listed here  **: The total number of genes analyzed in microarrays is not specified |
| Orellana et al., 2016 (Chile) | **24**  12 (+)  12 (-) | (+): 8-14  (-): 5-17 | (+): 58%  (-): 42% | Yes | Histopathology and rapid urease test | qRT-PCR / gastric biopsy | 1  (SLC5A8) | 0 |  |
|  | **94**  50 (+)  44 (-) | (+): 2 – 8.3  (-): 2 – 7.5 | (+): 42%)  (-): 43% | No | Stool antigen test | qRT-PCR / whole blood |  |  | - |
|  | **16**  11 (+)  9 (-) | < 5 |  |  |  |  |  |  |  |
| Ryszczuk et al., 2016 (Poland) | **44**  30 (+)  14 (-) | 5-18 | (+): 46%  (-) 50% | Yes | Histopathology and serology | Immunohistochemistry / gastric biopsy | 2  (BCL-2, EGFR) | 0 | - |
| Kassem et al., 2017 (Isrsel) | **278**  **99 (+)**  **179 (-)** | 11.2 ± 3.6 | 40% | Yes (99)  No (179) | Serology (serum IgG anti H. pylori and anti CagA) | ELISA / serum | 2  (Pepsinogen 1, Pepsinogen II) | 0 | - |
| Alvarez et al., 2018 (Brazil) | **50**  (+) 22  (-) 28 | 2-18 | 53% | Yes | Histopathology, rapid urease test,and PCR of gastric biopsy. | qRT-PCR / gastric biopsy | 0 | 1  (GATA-5^¢,^  TFF1) | ¢: Overexpressed in unmethylated samples of infected children compared to unmethylated samples of non-infected children; but no data about comparison of total samples between groups. |
| Cortés-Márquez et al., 2018 (Mexico) | **25**  16 (+)  9 (-) | 2-17 | 36% | Yes | PCR of gastric biopsies | qRT-PCR / gastric biopsy | 2  (miRNA-146a, miRNA-155) | 0 | - |
| Kienesberger et al., 2018 (Bangladesh) | **18**  (+) 13  (-) 5 | (+): 1-1.5  (-): 0.5-1.5 | NA | No | Serology | ELISA / serum | 1  (Pepsinogen I) | 0 | Cohort study from birth. Comparison of pepsinogen I levels was made between children who never became seropositive to *H. pylori* (controls) and children who became persistently seropositive. |

(+): *H. pylori* positive (-): *H. pylori* negative

N: Number of children included in study HP: *Helicobacter pylori*

No.: Number UBT: Urea breath test ND: Not determined

RIA: Radioimmunoassay ELISA: enzyme linked immunosorbent assay CLEIA: chemiluminescent enzyme immunoassay

NA: not available

*: When range of age is not available, mean ± SD is shown.
