# Supplementary material for: Gastric Damage and Cancer-Associated Biomarkers in Helicobacter pylori-Infected Children
Source: Front Microbiol. 2020 Feb 12;11:90. doi: 10.3389/fmicb.2020.00090 (PMC7029740; doi:10.3389/fmicb.2020.00090)
Supplement: Supplementary file 3 [file Table_2.docx]

**Supplementary Table 2. Studies on biomarkers depicting methylation status included in this review**

| **Reference (Country)** | **N (children)** | **Age (range in years)** | **Gender**  **(% female)** | **Abdominal Symptoms** | **HP status assessment** | **Methylation assessment method / samples** | **No. of biomarkers differentially methylated according to infectious status (details)** | **No. of biomarkers not differentially methylated according to infectious status (details)** |
| --- | --- | --- | --- | --- | --- | --- | --- | --- |
| Shin et al., 2011  (Korea) | **47**  (+) 27  (-) 20 | (+): 4-18  (-): 4-17 | (+): 40%  (-): 40% | Yes | Histopathology and rapid urease test | Bisulfite conversion, qPCR and sequencing / gastric biopsy | 7  (CALCA, CDH1, CRABP1, DAPK1, GRIN2B, TIMP3, TWIST1) | 5  (BR3C1, CYP1B1, HOX1, NEUROG1, SMAD9) |
| Alvarez et al., 2013(a) (Brazil) | **50**  (+) 22  (-) 28 | 8 ± 4 | NA | Yes | Histopathology and PCR of gastric biopsy | Bisulfite conversion and Methylation specific PCR / gastric biopsy | 2  (GATA-4, THBS1) | 1  (HIC1) |
| Alvarez et al., 2013 (b) (Brazil) | **50**  (+) 22  (-) 28 | 2-18 | 53% | Yes | Histopathology and PCR of gastric biopsy | Bisulfite conversion and methylation specific PCR / gastric biopsy | 0 | 2  (MGMT, MLH1) |
| Alvarez et al., 2018 (Brazil) | **50**  (+) 22  (-) 28 | 2-18 | 53% | Yes | Histopathology, rapid urease test,and PCR of gastric biopsy. | Bisulfite conversion and methylation specific PCR / gastric biopsy | 1 (GATA-5) | 0 |

(+): *H. pylori* positive

(-): *H. pylori* negative

N: Number of children included in study

HP: *Helicobacter pylori*

No.: Number

NA: Not available
