# Supplementary material for: Gastric Damage and Cancer-Associated Biomarkers in Helicobacter pylori-Infected Children
Source: Front Microbiol. 2020 Feb 12;11:90. doi: 10.3389/fmicb.2020.00090 (PMC7029740; doi:10.3389/fmicb.2020.00090)
Supplement: Supplementary file 4 [file Table_3.docx]

**Supplementary Table 3**

**Brief description of the genes and proteins identified in this review.**

| **Gene/Protein (alphabetical order)** | **Biological Function** | **References** |
| --- | --- | --- |
| **β-catenin** | Multifunctional E-cadherin-binding protein, regulating cell-to-cell adhesion, and transcriptional regulator in the Wnt signaling pathway, regulating proliferation, stem cell maintenance and homeostasis in normal gastric mucosa. | Ebert et al., 2003; Chiurillo 2015. |
| **Bcl-2** | The B cell lymphoma 2 family of proteins constitutes the mitochondrial or intrinsic apoptotic pathway, and can be classified into 3 functional groups: anti-apoptotic proteins, such as BCl-2 itself, pro-apoptotic effectors, or pro-apoptotic activators. | Czabotar et al., 2014 |
| **C-MYC** | Belongs to the MYC proto-oncogene family, encoding transcription factor proteins implicated in regulation of cell proliferation/differentiation and apoptosis. | McMahon, 2014 |
| **CALCA** | Calcitonin Related Polypeptide Alpha encodes the polypeptides procalcitonin (PCT) and the PCT gene-related peptide-α (proCGRPα); CALCA’s role in human cancer is not completely understood. | Christ-Crain and Müller, 2008 |
| **CCL18** | Chemokine- CC motif- ligand 18 is mainly produced by monocyte-derived cells, involved in inflammation and generation of the immune response. In cancer, its overexpression has been reported in several cancer tissues including GC, associated with poor prognosis. However, there is contradictory evidence in GC, as other authors have reported a potential tumor-suppressing role by stimulating antitumoral immunity. | Schutyser et al., 2005; Leung et al., 2004; Huang et al., 2018. |
| **CDH1** | Cadherine 1 plays a role in cell-to-cell adhesion and its mutations are associated with several solid tumors, including GC. | Melo et al., 2017; Caggiari et al., 2017; van der Post and Carneiro, 2017 |
| **COX-2** | An enzyme responsible for prostaglandin synthesis, its expression is induced at sites of inflammation and abnormal cellular proliferation, unlike COX-1 expression, which is constitutive in normal gastric tissue. It is overexpressed in several cancers, promoting cancer development by induction of apoptotic resistance, proliferation, angiogenesis, invasion, and metastasis, but in some cases acts as an anticancer enzyme | Vane et al., 1998; Hashemi Goradel et al., 2019. |
| **CRABP1** | Cellular retinoic acid-binding protein 1 modulates Retinoic Acid (RA) signaling and its role in carcinogenesis is controversial. It promotes apoptosis of cancer cells *in vitro*, but may also have pro-metastatic and pro-tumorogenic activity in mesenchymal tumors. | Persaud et al., 2016; Kainov et al., 2014. |
| **CXCL13** | Chemokine -CXC motif- ligand 13 is a B-cell attracting chemokine expressed by stromal cells, involved in tumor cell proliferation, migration and associated with poor prognosis in several neoplasms including GC, where intra-tumoral CXCL13 over expression is associated with greater tumor diameter and overall reduced survival. | Wei et al., 2016; Fan et al., 2017. |
| **CXCL9, 10, 11** | Chemokines –CXC motif- ligand 9, 10, 11 are produced mainly by monocytes, endothelial cells, fibroblasts in response to IFN gamma , and shares functions as selective ligands for CXCR3 receptor located in immune cells. In cancer, the CXCL9, 10, 11-CXCR3 axis is involved in promoting antitumoral immune response in a paracrine axis, but also in tumor growth and metastasis in an autocrine axis. | Tokunaga et al., 2018. |
| **DAPK1** | Death associated protein kinase 1 is a positive mediator of interferon-γ, inducing apoptosis and acting as a tumor suppressor gene in normal tissue; however, in cancer tissue this protein stimulates cell proliferation and angiogenesis. | Li et al., 2017 |
| **DC-SIGN** | C-type lectin that recognizes carbohydrates from microorganisms, and also mediates dendritic cell adhesion and migration. An oncogenic function has been described, promoting metastasis and immune evasion . | Jiang et al., 2014, Wu et al., 2014 |
| **EGFR** | This epidermal growth factor receptor is a tyrosine-kinase receptor involved in cell survival, proliferation, differentiation, and migration by activation of ERK/MAPK, phosphatidyl-inositol-3-kinase (PI3K), and the JAK/STAT pathway. | Wee and Wang, 2017. |
| **Gastrin** | Gastrointestinal peptide normally produced by antrum G cells in response to stimulus like food, decreased acid, and gastric distension. Stimulates acid production and regeneration of gastric epithelium. Long-term hypergastrinemia contributes to gastric carcinogenesis. | Smith et al., 2017, Waldum et al., 2017 |
| **GATA-4 and 5** | Belong to GATA family of transcription factors. They act as regulatory proteins guiding the development and differentiation in endoderm-derived organs, being considered markers of terminal differentiation in the gastrointestinal epithelium, and tumor suppressor genes. | Akiyama et al., 2003; Jonckheere et al., 2012; Zheng and Blobel, 2010. |
| **GRIN2B** | GRIN2B codifies the ionotropic glutamate receptor NMDAR2B, described as expressed on the surface of cancer cells and as a potential therapeutic target. | Deutsch et al., 2014 |
| **Ki-67** | A nuclear protein associated with cell proliferation; it is expressed during all active phases of the cell cycle (G1, S, G2, and M phases), but is not detected during the resting phase (G0), resulting in its wide use as a cell proliferation marker, including cancer tissue. | Li et al., 2015 |
| **LCN2** | Lipocalin 2 or Neutrophil gelatinase associated lipocalin is a secreted glycoprotein that transports small hydrophobic ligands and is overexpressed in several benign and malignant diseases. | Akelma et al., 2012; Chassaing et al., 2012; Chakraborty et al., 2012 |
| **miRNA-146a** | miRNA involved in the regulation of innate immune response and pathogen-induced inflammatory response. Its role in carcinogenesis is unclear, promoting cell proliferation and metastasis or acting as tumor suppressor in different cells and tissues. | Li et al., 2010; Hou et al., 2012 |
| **miRNA-155** | miRNA indispensable for the immune response, which deregulation contributes to the development of chronic inflammation, autoimmunity, cancer, and fibrosis. Both upregulation and downregulation have been associated with carcinogenesis. | Tili E et al., 2009; Higgs et al., 2013 |
| **MUC5AC** | Mucins are extracellular glycoproteins produced by epithelial tissue and involved in protection, lubrication and hydration of the external surface of human epithelial tissue layers. During neoplastic transformation, expression of specific mucins may be reduced. The mucin proteins (apomucins) MUC 2, MUC5AC, MUC6 (secreted), and MUC1 (transmembrane) are normally expressed in gastric tissue. | Corfield et al., 2001 |
| **P21** | p21 protein is an inhibitor of cyclin-dependent kinase, which arrests the cell cycle, acting as a tumor suppressor protein. However, there is increasing evidence as to p21’s oncogenic role, acting as an anti-apoptotic agent and acquiring pro-oncogenic properties in a p53-deficient cell environment | Bieging et al., 2014; Georgakilas et al., 2017. |
| **p53** | Protein acts as a transcription factor, which up regulates p21 expression, an inhibitor of cyclin-dependent kinase that arrests the cell cycle at the G1 phase. | Bieging et al., 2014 |
| **PGI/ PGII** | Pepsinogens (PGs) are precursors of pepsin, an enzyme produced by gastric chief cell, and are classified in PG I (produced by chief and mucous cells in the fundic glands) and PG II (also produced by cells in the pyloric and Brunner’s glands). PGs are secreted into the gastric lumen, and a minor fraction can be found in the serum, allowing their use as biomarkers of the functional and anatomical integrity of gastric mucosa. Changes in expression levels occur in both inflammation and neoplasms of gastric tissue. | Shiota et al., 2014. |
| **PIM** | The proviral integration site of Moloney virus kinases are serine-threonine kinases responsible for cell cycle regulation, antiapoptotic activity and the homing and migration of receptor tyrosine kinases mediated via the JAK/STAT pathway. | Blanco-Aparicio and Carnero, 2013 |
| **PPAR-Y** | Nuclear receptor expressed in adipocytes and epithelial cells. It regulates fatty acid metabolism, and the expression of pro-inflamatory cytokines. It can induce apoptosis, and synthetic agonists inhibit cell proliferation. | Haruna et al., 2008; Vella et al., 2017 |
| **REG3A** | Regenerating islet-derived 3 alpha is a member of the REG protein family, which in gastric tissue are growth and anti-apoptotic factors. REG3A, also named human hepatocarcinoma-intestine-pancreas or human pancreatitis-associated protein, is normally expressed in the pancreas and small intestine, and has been shown to be involved in the development of several cancers, including hepatocellular carcinoma, colorectal cancer and GC. | Parikh et al., 2012; Ye et al., 2016; Cavard et al., 2006 |
| **SLC5A8** | The solute carrier family 5 member 8 encodes a monocarboxylate transporter located in several tissues and organs, including the gastrointestinal tract. It acts as a cancer-suppressor gene by facilitating the entrance of short chain fatty acids into the cell, inhibiting inflammation, promoting cell differentiation, stimulating cell cycle arrest and inducing apoptosis. Decreased expression of SLC5A8 has been described in several human tumors, including GC. | Ueno et al., 2004; Ganapathy et al., 2008; Brim et al., 2011. |
| **THBS1** | Thrombospondin 1 has an anti-angiogenic effect in several solid tumors acting as a tumor suppressor gene. In advanced tumors, THBS-1 may function as an adhesive protein or a modulator of extracellular proteases in order to promote tumor invasion. | Maeda et al., 2001, Grossfeld et al., 1997; Kazerounian et al., 2008. |
| **TIMP1 and TIMP3** | Matrix metalloproteinase are zinc-dependent proteases involved in a number of physiological processes, tumor invasion, and in the development of metastatic disease through degradation of the extracellular matrix. Their activity is regulated *in situ* by endogenous inhibitors, such as tissue inhibitor metalloproteinases (TIMP). TIMP1 levels are high in several aggressive tumors and are associated with poor outcomes, possibly explained by MMPs-independent functions like inhibition of apoptosis, induction of angiogenesis and direct stimulation of cell proliferation. In contrast to TIMP1, increased expression of TIMP3 results in the suppression of tumor growth and angiogenesis, whereas decreased expression leads to invasion and metastasis. | Kessenbrock et al., 2010; Jackson et al., 2017; Anand-Apte et al., 1996; Ahonen et al., 1998. |
| **TWIST-1** | Belongs to the basic-helix-loop-helix family of transcription factors involved in epithelial–mesenchymal transition processes, and plays an important role in cell migration during embryonic development, tumor invasion and metastasis. | Izadpanah et al., 2014; Yang et al., 2007 |
| **VCAM-1** | Vascular cell adhesion molecule-1 acts as cell adhesion molecule normally expressed in endothelial cells, which expression is promoted in chronic inflammatory conditions in cells as macrophages, dendritic cells and tumor cells. Plays a role in angiogenesis and metastasis in several tumors as breast, lung, colorectal and gastric cancer | Kong et al., 2018 |
|  |  |  |

GC: Gastric cancer
